# A novel *EDA1* missense mutation in X-linked hypohidrotic ectodermal dysplasia

**DOI:** 10.1097/MD.0000000000019244

**Published:** 2020-03-13

**Authors:** Xu Wang, Zhiyu Zhang, Shuo Yuan, Jiabao Ren, Hong Qu, Guozhong Zhang, Wenjing Chen, Shushen Zheng, Lingqiang Meng, Jiuping Bai, Qingqing Du, Dongru Yang, Wenjing Shen

**Affiliations:** a Department of Prosthodontics, School and Hospital of Stomatology, Hebei Medical University and Hebei Key Laboratory of Stomatology, Shijiazhuang; b College of Life Sciences, Peking University, Beijing; c College of Forensic Medicine, Hebei Medical University; d Department of Periodontics; e Department of Orthodontics, School and Hospital of Stomatology, Hebei Medical University & Hebei Key Laboratory of Stomatology, Shijiazhuang; f Xingtai Medical College, Xingtai; g Department of Oral and Maxillofacial Surgery, School and Hospital of Stomatology, Hebei Medical University and Hebei Key Laboratory of Stomatology, Shijiazhuang, PR China.

**Keywords:** ectodysplasin A, hypohidrotic ectodermal dysplasia, missense mutation, tooth abnormalities

## Abstract

A mutation in the epithelial morphogen gene ectodysplasin-A1 (*EDA1*) is responsible for the disorder X-linked hypohidrotic ectodermal dysplasia (XLHED), the most common form of ectodermal dysplasia. XLHED is characterized by impaired development of hair, eccrine sweat glands, and teeth. This study aimed to identify potentially pathogenic mutations in four Chinese XLHED families.

Genomic DNA was extracted from the peripheral blood and sequenced. Sanger sequencing was used to carry out mutational analysis of the *EDA1* gene, and the three-dimensional structure of the novel mutant residues in the EDA trimer was determined. Transcriptional activity of NF-κB was tested by Dual luciferin assay.

We identified a novel *EDA1* mutation (c.1046C>T) and detected 3 other previously-reported mutations (c.146T>A; c.457C>T; c.467G>A). Our findings demonstrated that novel mutation c.1046C>T (p.A349 V) resulted in XLHED. The novel mutation could cause volume repulsion in the protein due to enlargement of the amino acid side chain. Dual luciferase assay revealed that transcriptional NF-κB activation induced by XLHED EDA1 protein was significantly reduced compared with wild-type EDA1.

These results extend the spectrum of *EDA1* mutations in XLHED patients and suggest a functional role of the novel mutation in XLHED.

## 1 Introduction

Hypohidrotic ectodermal dysplasia (HED) is the one of the most common forms of ectodermal dysplasias (EDs), a group of disorders characterized by sparse hair, oligodontia, and reduced sweating.^[1–3]^ It is caused by mutations in any of the ectodysplasin A (EDA) pathway genes.^[4,5]^ Cui and Schlessinger^[6]^ suggested that mutations in *EDA*, *EDAR*, or *EDARADD* genes are the causes of HED in humans. A previous study demonstrated that the X-linked recessive inheritance pattern constitutes the majority of HED cases, and that the others are dominated by autosomal dominant or recessive inheritance.^[7]^ According to Trzeciak,^[8]^ there have been 345 reported cases of HED, of which 206 are due to *EDA1* gene mutations. As at 2017, the Human Gene Mutation Database (HGMD Professional 2017.2) had registered 314 mutations in the *EDA1* gene. ^[9]^ Nucleotide mutations in the *ectodysplasin A* (*EDA*) gene, which is located in Xq12-q13.1,^[2,10]^ are the main causes of XLHED (OMIM: 305100) features.^[2,11,12]^ The EDA signaling pathway plays an important role in embryonic ectodermal development.^[13,14]^ Mutations in genes involved in this pathway disturb the interaction between surface-located epithelial cells and the underlying mesenchyme^[15]^ during embryonic development, which causes alterations of the initiation, formation, and differentiation of skin appendages. *EDA1* is the only gene known to be associated with XLHED, ^[16]^ and 95% of individuals with HED have the X-linked form. The genes *EDAR* and *EDARADD* are known to be associated with both autosomal dominant and recessive forms of HED, and mutations in these genes account for the other 5% of HED.^[9]^ In the present study, we report a novel missense mutation (EDA1 c.1046C>T), as well as 3 previously-reported missense mutations in four Chinese Han XLHED families.

## 2 Materials and methods

### 2.1 Ethical approval

The protocol of this study was approved by the Institutional Review Board of the School and Hospital of Stomatology, Hebei Medical University. All aspects of this study were carried out following the approved guidelines. All participants or their guardians signed written informed consent. This study followed the recommendations of the STROBE guidelines (https://www.strobe-statement.org/index.php?id=strobe-home).

### 2.2 DNA sample collection and extraction

The probands were patients at the School and Hospital of Stomatology, Hebei Medical University. Physical examination was performed as thoroughly as possible by two dentists, and panoramic radiograph film was used to evaluate the development of tooth and alveolar bone. Other family members were also interviewed and examined. Peripheral blood samples were collected using EDTA as anticoagulant. DNA was extracted from leukocytes using standard proteinase-K phenol chloroform methods (E.Z.N.A. Blood DNA Midi Kit, Omega, GA) and stored at −20°C.

### 2.3 PCR amplification and mutation screening

The primers used to amplify the eight coding exons of the *EDA* gene in PCR were referred to from Song et al.^[17,18]^ PCR reactions were elicited in a total volume of 50 μl, each containing 100 ng DNA, 4 μl dNTPs, 5 μl 10× TransStart *Taq* Buffer, 0.2 μM each primer, and 1.25 U TransStart *Taq* DNA Polymerase (Thermo Fisher, USA). After denaturing at 95°C for 5 min, amplification was carried out as follows: 35 cycles at 95°C for 30 seconds, 60°C for 30 seconds, 72°C for 30 seconds, and finally 72°C for 7 minutes. Amplification was tested by agarose gel electrophoresis and DNA was sequenced by the Beijing Genomics Institute, Beijing, China. The nucleotide sequence was analyzed using the BLAST database of the National Center for Biotechnology Information (NCBI). We identified the nucleotide variant in the *EDA* gene and 100 unrelated population-matched controls.

### 2.4 Mutant protein modeling

A molecular model of the EDA1 protein was generated based on the structure PDB ID: 1RJ7 (https://www.ncbi.nlm.nih.gov/Structure/pdb/1RJ7). The model of 1RJ7 was used to illustrate the effect of mutation on function. The model of EDA1 consists of 4 trimers, each of which consists of three identical monomers stacked together to form an active channel.

### 2.5 Luciferase assay

The methods for constructing the EDA1 expression vector (A349 V) and the luciferase assay were reported in a previous study.^[19]^ In brief, LS8 cells were co-transfected with three plasmids (pNF-κB-luc, EDA mutant, and pRL-TK) and cultured for 48 hours. Vector pCR3 and a wild-type EDA-encoding vector were used as negative and positive controls, respectively. Following culturing, firefly and Renilla luciferase activities were measured using a dual luciferase assay system (Promega Corporation, Madison, WI) on a Synergy 2 Multi-Detection Microplate Reader (BioTek, Winooski, VT). Firefly luciferase activity was standardized against Renilla luciferase control activity. Each experiment was performed in triplicate and repeated at least 5 times. Results were assessed for statistical significance using Student's *t* test (*P* < .05).

## 3 Results

### 3.1 Clinical manifestations

**Figure 1:**
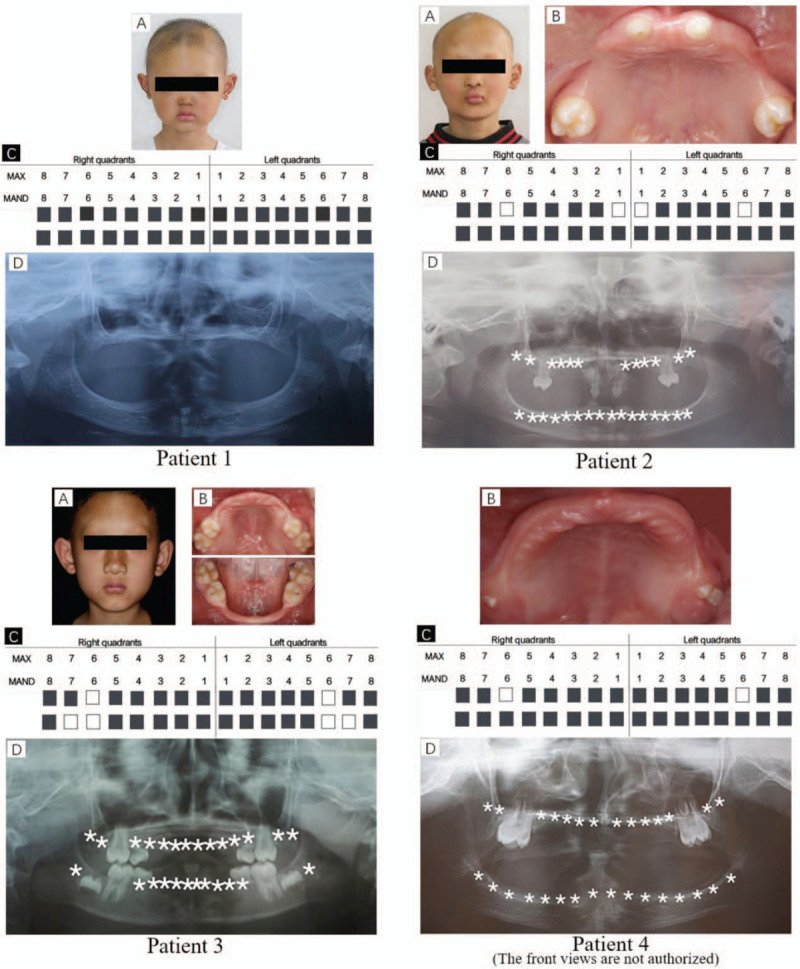
Characteristics of individuals with hypohidrotic ectodermal dysplasia (HED). (A) Positive image of probands; (B) Intraoral image of probands; (C) Legend of intraoral missing teeth of probands; (D) Panoramic radiography of probands.

Four patients that fulfilled the clinical diagnostic criteria of XLHED were investigated. According to the results of oral examination, oligodontia or anodontia were seen, with most patients’ teeth arranged symmetrically; the size and shape of the remaining permanent crowns were abnormal; facial examination also showed the characteristic facial features of a saddle-shaped nose, forehead protrusion, hypohidrosis and hypotrichosis, sparse eyebrows, no eyelashes, and dry skin (Fig. 1). There was no difference in mental development between the patients and age-matched controls.

### 3.2 Analysis of *EDA* gene mutations

**Figure 2:**
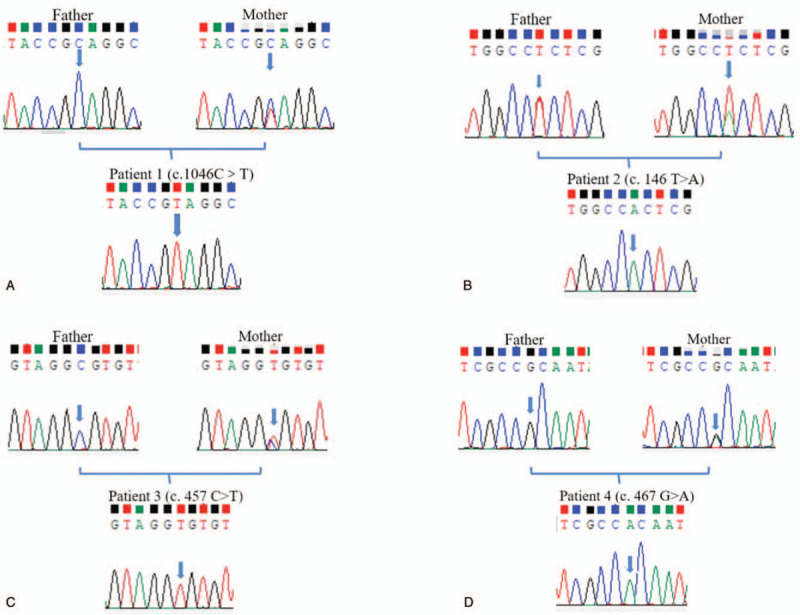
Sequencing results show *EDA1* mutations in four families. The four probands, affected with HED, were found to have missense mutation: (A) Patient 1: *EDA1* c.1046 C>T; (B) Patient 2: *EDA1* c. 146 T>A; (C) Patient 3: *EDA1* c.457 C>T; (D) Patient 4: *EDA1* c.467 G>A. Mothers of patient are all heterozygotes, fathers are all unaffected.

**Figure 3:**
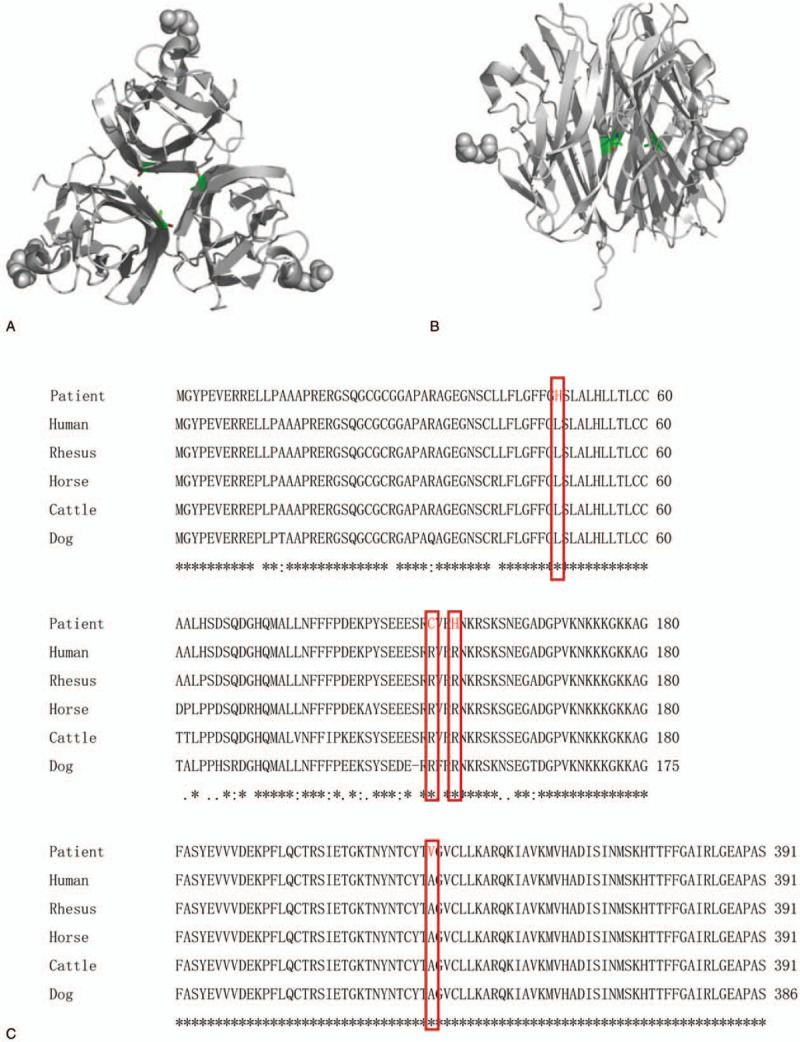
Protein structure of 3 monomers showing the conserved locations of the mutant sites in the EDA protein sequence. (A) Top view of the trimer of EDA; (B) side view of the trimer of EDA; (C) Sequence alignment results show that the four residues are conserved across five species: the mutant allele is boxed.

After diagnosis, we aimed to determine the causative gene. We screened the coding sequence of the *EDA* gene and identified four *EDA1* missense mutations, as well as a novel missense mutation (c.1046C>T) in an exon of *EDA1*. The mutation of 349 amino acid from GCA to GTA, located in the TNF homology domain, resulted in a change of amino acid from alanine to valine. Patient 1 had single-base mutation C>T in this locus, and his mother showed a hybrid doublet of C and T in this site. In contrast, the control showed a singlet of C, maintaining normal alignment. There was also a (c.1046C>T) mutation in exon 9 of the *EDA* gene in patient 1 and his mother was a carrier (Fig. 2A). Three of the four mutations found in sporadic HED patients have been reported in exons of *EDA1*: c.146T>A^[20]^ (Fig. 2 B); c.457C>T^[21]^ (Fig. 2C); and c.467G>A^[22]^ (Fig. 2D). The mothers of the patients were heterozygote and therefore carriers. The sites of these mutations are conserved among normal human, rhesus monkey, horse, cow, and dog protein sequences (Fig. 3C).

### 3.3 Protein structure of three monomers of an abnormal EDA trimer

The three-dimensional structure was modeled as PDBID: 1RJ7, which was used to illustrate the effect of mutation on function. Residue A349 was located on the interaction surface between the three monomers. The hydrophobic interaction of the three residues could play an important role in the formation of the trimer. When the A349 V mutation occurs (indicated using sticks in Fig. 3A and B), the side chain of the Val residue has two more CH_3_-groups than Ala. This may cause volume rejection due to enlargement of the side chain, which in turn may not be able to maintain the stability of the active structure of the trimer.

The L49H mutation occurs with a change from a hydrophobic residue to a positively-charged hydrophilic residue. The R153C mutation occurs with a change from a positively-charged hydrophilic residue to a uncharged hydrophilic residue. Although the R156H mutation does not change in physicochemical properties, the volume of the side chains shrinks, which may change with the surrounding residue. So it is speculated that they will have a significant impact on EDA activity.

### 3.4 *XLHED-EDA1* mutation impairs the transcriptional activation of NF-κB

**Figure 4:**
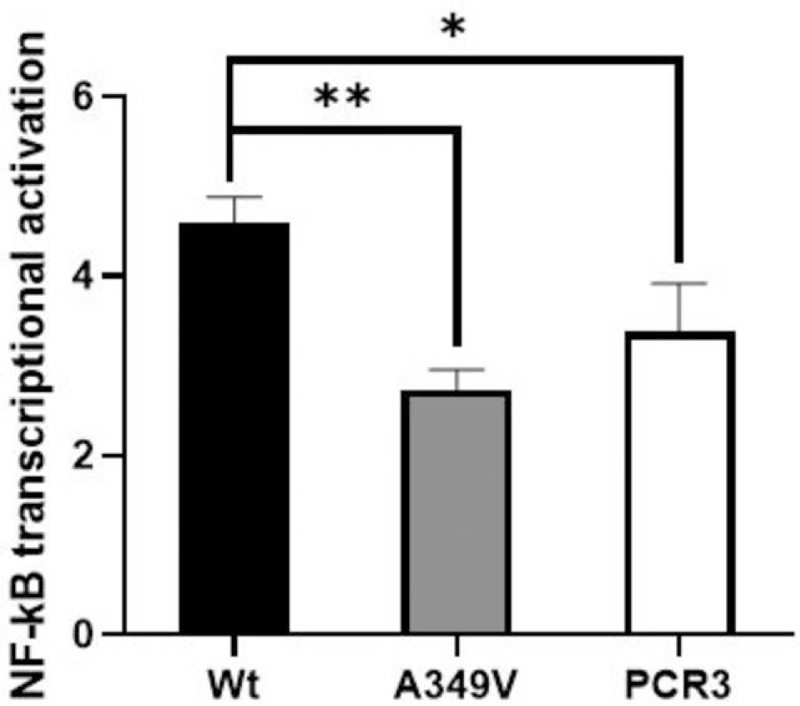
Effect of *XLHED-EDA1* mutation on the transcriptional activation of NF-κB by dual luciferase assay. It shows that transcriptional NF-κB activation induced by mutant EDA1 protein (A349 V) was significantly reduced compared to wild-type EDA1. ^∗^*P* < .01; ^∗∗^*P* < .001; Wt: wild-type.

*EDA1* is expressed in dental epithelium during tooth development.^[23]^ To analyze the mechanism of how *EDA1* mutations affect the function of dental epithelial cells in vitro, we used epithelium-derived ameloblast cell line LS8. It has been established that binding between EDA1 and its receptor induces activation of the Eda/Edar/NF-κB signaling pathway.^[19,24]^ Our results indicated that XLHED-EDA1 mutant protein A349 V significantly impairs transcriptional activation of NF-κB compared with wild-type EDA1 in LS8 cells (Fig. 4).

## 4 Discussion

XLHED is the most common type of over 200 different ectodermal dysplasias.^[9]^ As illustrated by Kere et al and Zhang et al, mutations located in the *EDA* gene are the most common causes of XLHED, with a prevalence rate of 63% to 95%. In this study, we found four missense mutations (p. A349 V, p. R156H, p. L49H, and p. R153C)^[20–22]^ in the *EDA* gene that have previously been found to be responsible for XLHED, but the mutation detected in exon 9 (p. Ala349Val) has never been reported before. According to previous reports of *EDA* gene mutation, 98% of mutations occurred in exons 1, 3, 5, 8, and 9, which constitute the most important domain of *EDA*.^[22,25]^ In our research, the mutational sites are located in exons 1, 3, and 9 respectively, confirming the above point.

Furthermore, sequence alignment results show that the four mutation locations are conserved in 6 other species.^[26,27]^ Using bioinformatics analyses, we showed that the novel mutation (A349 V) was located at the surface of the protein, influencing the stability of the homotrimer and causing the XLHED-EDA1 protein to lose all receptor binding capability.^[19,28]^ It seems that in the presence of XLHED, tooth agenesis appears to completely block signaling through the NF-κB pathway.^[19]^ NF-κB suppression results in severe defects at the early stages of epidermal appendage development, with an epidermal phenotype that is analogous to HED in humans and identical to the phenotypes of Eda^−/−^, Edar^−/−^, or crinkled mice.^[29]^ Our study showed that XLHED-EDA1 mutant protein (A349 V) significantly impairs transcriptional activation of NF-κB compared with wild-type EDA1 in LS8 cells. The results from the current study strongly support our previous conclusions.^[19]^ Therefore, the *EDA* gene is critically involved in the process of ectodermal development and thus the various congenital anomalies, including hypoplasia or absence of eccrine sweat glands, hair and teeth. The phenotypic features present in patients with HED have proved to be very useful in clinical diagnosis, however there is no direct correlation between the severity of the disease and the type of mutation. ^[30,31]^

*EDA* is an important regulatory gene for controlling ectodermal morphogenesis in HED patients, and the reported mutations normally include missense mutations, nonsense mutations, splice junction mutations, and molecular transposition. Mutations in EDA proteins ultimately lead to HED by blocking binding to the specific receptor^[19,21,28]^ and inhibit the downstream NF-κB signaling pathway.^[19]^ As the phenotypic penetrance of the mutations is very variable, further studies are necessary to show a more direct genotype–phenotype correlation for HED and to fully understand the mechanism of pathogenesis of HED.

## 5 Conclusion

Our findings demonstrate that a novel c.1046C>T mutation (p.A349 V) resulted in XLHED. The novel mutation could cause volume repulsion in the protein due to enlargement of the amino acid side chain. The mutant protein (A349 V) significantly impairs transcriptional activation of NF-κB. This study expands the known *EDA* mutation spectrum and could help in gene detection, pregnancy counseling, and fetus diagnosis, which help in disease status prediction in X-linked *EDA* families.

## Acknowledgments

We sincerely thank all the subjects and medical staff involved in this study for their help in sample collection, diagnosis, and analysis. We are also very grateful to the College of Forensic Medicine of Hebei Medical University and Hebei Key laboratory of forensic medicine for providing lab instruments and technical support.

## Author contributions

**Conceptualization:** Wenjing Shen, Dongru Yang.

**Data Curation:** Wenjing Shen, Xu Wang, Zhiyu Zhang.

**Funding acquisition:** Wenjing Shen.

**Methodology:** Guozhong Zhang, Qingqing Du.

**Resources:** Guozhong Zhang, Shushen Zheng.

**Supervision:** Wenjing Shen.

**Software (Reconstruction of three-dimensional structure of EDA protein):** Hong Qu.

**Clinical cases collection:** Jiabao Ren, Wenjing Chen, Shuo Yuan, Lingqiang Meng, Jiuping Bai, Shushen Zheng.

**Prominent researcher:** Xu Wang, Zhiyu Zhang.

**Writing – original draft:** Wenjing Shen, Xu Wang, Zhiyu Zhang.

**Writing – review & editing:** Wenjing Shen, Zhiyu Zhang.
